# Associations between medical students’ beliefs about obesity and clinical counseling proficiency

**DOI:** 10.1186/s40608-018-0222-4

**Published:** 2019-02-04

**Authors:** Victoria Fang, Colleen Gillespie, Ruth Crowe, Dennis Popeo, Melanie Jay

**Affiliations:** 1 Department of Medicine, New York University School of Medicine, New York, NY USA; 2 Department of Psychiatry, New York University School of Medicine, New York, NY USA; 3 Departments of Medicine and Population Health, New York University School of Medicine, New York, NY USA; 4 New York Harbor Veterans Health Affairs, New York, NY USA

**Keywords:** Obesity attitudes, Obesity counseling, Obesity education

## Abstract

**Background:**

Despite evidence that biological and genetic factors contribute strongly to obesity, many healthcare providers still attribute obesity more to controllable behavioral issues rather than factors outside a person’s control. We evaluated whether medical school students’ beliefs about obesity correlate with ability to effectively counsel patients with obesity.

**Methods:**

Clerkship-year medical students at NYU School of Medicine completed an Objective Structured Clinical Experience (OSCE) that tests ability to effectively counsel standardized actor-patients with obesity. We surveyed these students to evaluate their beliefs about the causes of obesity and their attitudes towards people with obesity. We analyzed correlations between student beliefs, negative obesity attitudes, and OSCE performance.

**Results:**

The response rate was 60.7% (*n* = 71). When asked to rate the importance of individual factors, students rated controllable factors such as unhealthy diet, physical inactivity, and overeating as more important than genetics or biological factors (*p* < 0.01). Believing obesity is caused by uncontrollable factors was negatively correlated with obesity bias (*r* = − 0.447; *p* < 0.0001). Believing that obesity is caused by factors within a person’s control was negatively correlated with counseling skills (*r* = − 0.235; *p* < 0.05).

**Conclusions:**

Attribution of obesity to external factors correlated with greater ability to counsel patients with obesity, suggesting that educating providers on the biological causes of obesity could help reduce bias and improve provider care.

## Background

Obesity is one of our greatest public health challenges. About 36% of American adults have obesity, which is associated with multiple harmful health outcomes that include diabetes, heart disease, stroke, and several cancers. Moreover, obesity is associated with a lower quality of life, negative mental health consequences, and increased all-cause mortality [1].

Most of the general American public believe that obesity is caused by controllable lifestyle factors rather than biological causes or other external factors. For example, 75% say that Americans are overweight due to “not getting enough exercise,” and 59% attribute obesity to the “lack of willpower over eating.” In contrast, only 32% and 50% believe that “genetics and hereditary factors” and the “kinds of foods marketed at restaurants and groceries” are important causes of being overweight, respectively [2]. Numerous studies, however, demonstrate that genetics and heredity are a major factor in determining who within society will have obesity [3–9]. Two particularly compelling studies showed that people adopted into new families showed very little body mass index correlation with their adopted parents, but a strong correlation with their biological parents and siblings [3, 5]. These and other studies conclude that the heritability index of obesity is between 0.40 to over 0.70 [10]—nearly the same as that of height and higher than for heart disease or breast cancer. Other environmental factors such as increased portion sizes, easy access to calorie dense foods, increased convenience of eating at restaurants, marketing encouraging food consumption, more sedentary life styles encouraged by the prevalence of television/computer/cellphone screens, a more sedentary occupational landscape, poor food policies, lack of access to healthy food products, and even seemingly unrelated stressors such as psychosocial and financial stress can increase the risk of having obesity [11–13]. The evidence on the heritability of obesity and the abundance of uncontrollable environmental factors suggests that obesity results primarily from factors outside an individual’s control.

Additionally, stigmatization of people with obesity as lazy, weak-willed, unintelligent, and unsuccessful is widespread in the US and around the world [14, 15]; unlike stigma against other marginalized groups, these biases are, unfortunately, more socially acceptable. Much of this bias stems from the belief that obesity is within a person’s control rather than a result of uncontrollable factors. The perception that obesity is caused by controllable factors is a good predictor of negative bias towards people with obesity, whereas those who believe that obesity is largely out of a person’s control harbor less negative attitude towards persons with obesity [15, 16]. Medical students who attribute obesity to behavioral causes also have more negative biases towards patients with obesity [17–19].

The widespread attribution of obesity to controllable behaviors and the prevalence of anti-fat stigma can have negative impacts on the health care of patients with obesity. There is a negative correlation between the perception of personal responsibility and feelings of likability; stigmatized medical conditions are less likely to evoke sympathy, empathy, and intentions to help [20]. Indeed, there is evidence that perceived anti-fat bias in health care professionals decreases the likelihood that patients will seek medical care [21–25]. Obesity stigmatization also has negative consequences on the psychological and physical health of people with obesity and has been shown to negatively impact a person’s ability to lose weight [21, 26–29].

Several studies have shown that healthcare providers have anti-fat biases [22, 30–33] and others have reported that patients with obesity report feel stigmatized by their providers [25, 34, 35]. Few studies, however, have objectively evaluated whether beliefs and attitudes about obesity affects communication and counseling skills in treating patients with obesity.

An important set of counseling skills include the 5As counseling strategy, which has been recommended by the US Preventive Services Task Force for office-based counseling and has been useful in areas such as smoking cessation [36] and weight loss [37, 38] counseling. This framework guides providers to 1) assess behavioral risks and factors in behavioral change, 2) advise patients on behavioral changes, 3) agree on appropriate treatment goals, 4) assist patients in achieving goals, and 5) arrange for follow up and ongoing support. The 5A’s counseling strategy can be used by physicians to provide intensive behavioral therapy for obesity, which has been reimbursable by the Center for Medicare & Medicaid Services since 2011 [38].

In this study, we evaluated whether medical students’ beliefs about causes of obesity correlate with negative biases towards people with obesity, and we determined whether these beliefs and biases are associated with students’ ability to communicate with and counsel patients with obesity as assessed in a standardized patient encounter using a behaviorally anchored checklist.

## Methods

### Participants and implementation

Clerkship year students (years 1.5 to 2.5) at NYU School of Medicine completed a 3-day interclerkship intensive (ICI) program entitled “Fostering Change in Our Patients.” Among other topics, students had one full day of lectures and discussions about nutrition, obesity physiology, weight management, and disordered eating. Students then participated in an Objective Structured Clinical Experience (OSCE) during which they interviewed and counseled a standardized patient-actor about weight management. The student’s communication and counseling proficiency was evaluated by the standardized patients within several domains. At the conclusion of the ICI curriculum, students completed a survey about their beliefs about obesity and their attitudes towards persons with obesity. Survey responses were linked to OSCE performance for students who had provided consent for their routinely collected educational data to be used for medical education research as part of an NYU IRB-approved medical student research registry.

The sample of students who completed the OSCE case and consented to including their data in the Medical Education Research Registry (*n* = 117) did not differ significantly from the entire class (Class of 2019, *n* = 151) in terms of gender distribution (study sample = 55% female vs full class = 53% female, Chi Square = .08, *p* = .78), % under-represented minority status (study sample = 26% URM vs full class = 23% URM, Chi-Square = .22, *p* = .64), or mean age (study sample = 22.70, SD = 2.1 vs full class = 22.57, SD = 1.9; t-test = .54, *p* = .59). Of the 117 students who had consented to the medical student registry and completed the OSCE, 71 responded to the survey (61%). Response rates did not differ by gender.

### OSCE case and assessment of clinical skills

The OSCE case focused on lifestyle interventions for chronic disease management (diabetes and hypertension.) The standardized patient (SP) actors were middle-aged women with a self reported body mass index over 30 kg/m^2^, thus meeting criteria for having obesity. Students were advised that the patient had scheduled an office visit to discuss diet and weight loss, and asked to elicit a history and to counsel the patient accordingly. As part of the case, the standardized patient shared a food diary. Students’ proficiency in core communication skill performance was assessed by the standardized patient using an itemized rubric across several domains, including information gathering, relationship building, use of 5A’s counseling strategies [37], and patient activation. Within each domain, the item was rated by the SP as “not done (0 points),” “partly done (1 point),” or “well done (2 points)” with behavioral descriptors describing each of these response options. Points were summed across items within each domain. Overall professionalism and communication skills were rated on a 0–3 scale, a score of 3 indicating that the student was “Completely professional” or would be “highly recommended” to a friend or family member, respectively.

### Survey

Questions surveying student attitudes and beliefs were taken from the literature on obesity attitudes and physician competency in counseling patients with obesity [16, 31, 39–43]. Questions elicited student beliefs about the causes of obesity (Table 2) and their attitudes towards people with obesity (Table 3). Students were asked to rate the importance of various factors in contributing to obesity (i.e., “overeating,” “genetics or biological factors,” or “lack of willpower”). Each factor was scored using a 4-point Likert Scale (1, Not important; 2, somewhat important; 3, moderately important; 4, very important). Then, students were asked to self-assess their explicit bias towards people with obesity (i.e., “I feel uncomfortable when examining an obese patient.” or “Obese individuals don’t make good decisions.”) on a 4-point Likert-type Scale (1, strongly disagree, 2, somewhat disagree; 3, somewhat agree; 4, strongly agree).

### Analyses

Survey items were first analyzed by descriptive statistics for frequency distributions, mean, and SD. Differences in students’ ratings for each factor contributing to obesity were determined by one-way ANOVA followed by Tukey’s test to correct for multiple comparisons (Table 2). Items within each section of the survey were grouped and measured for internal consistency (Tables 2 and 3; Cronbach’s alpha 0.687 to 0.767). Bivariate two-tailed Spearman’s correlations were calculated to determine associations between bias measures (Table 4). For OSCE analysis, each domain was evaluated as a family of items and measured for internal consistency (Table 1, Cronbach’s alpha 0.638 to 0.777). Bivariate two-tailed Spearman’s correlations were calculated to determine associations between OSCE performance domains versus their beliefs and attitudes about people with obesity (Table 5). Finally, we used hierarchical regression to identify the contribution of external attribution of obesity to the ability of students to effectively counsel patients while controlling for the basic communication skills of information gathering. SPSS was used to conduct statistical analyses and Prism was used for graphing.

**Table 1:** OSCE Assessment Domains and Items

OSCE Skills	Skill parameters	Cronbach’s alpha
Communication skills: Information gathering	Used appropriate questions	0.664
	Managed the narrative flow	
	Allowed you to talk without interrupting	
	Clarified information by repeating to make sure you understood on an ongoing basis	
	Communicated concern or intention to help	
Communication skills: Relationship building	Non verbal behavior enriched communication (eye contact, posture)	0.657
	Acknowledged your emotions/feelings appropriately	
	Was accepting (nonjudgmental)	
	Used words patient understood and/or explained jargon.	
5As counseling strategy Note: “Arrange” parameter was not assessed in this OSCE.	Assessed how much weight you wanted to lose and discussed how much you should lose.	0.638
	Assessed motivation and/or importance to make changes to lose weight.	
	Assessed confidence in ability to make changes to lose weight.	
	Allowed patient to explain reasons for current dietary choices and/or what dietary changes she would be willing to make	
	Assessed physical activity (dancing, walking) and interest in increasing physical activity	
	Discussed possible specific diet, exercise, self-monitoring goals	
	Enlisted me in prioritizing a few specific goals (collaborative goal-setting)	
	Explored barriers or obstacles to achieving goals	
Patient activation	How much did this visit help me understand the nature of my problem/health condition	0.777
	How much did this visit make you want to change your behavior (engage in the recommended behavior)?	
	How much did this visit make you feel that you would be able to make the recommended changes/take recommended actions?	
Overall reccomendation	Overall, how would you rate this medical student’s professionalism?	0.669
	Would you recommend this medical student to a friend or family member for his/her overall communication skills?	
Total OSCE score	Sum of scores from all parameters	n/a

Each domain was evaluated as a family of items and measured for internal consistency

## Results

### Causes of obesity and attitudes towards those with obesity

Ratings for nine factors that may contribute to obesity are listed in Table 2 in ascending order of the mean student ratings, with items falling under the category of “within a person’s control” shaded in gray. Unhealthy diet (*p* < 0.0001), physical inactivity (*p* = 0.0004), or overeating (*p* = 0.003) were all rated significantly more important than genetics or biological factors as a cause of obesity. More than half of medical students rated unhealthy diet (62.0%), physical inactivity (56.3%), and overeating (52.1%) as very important contributors to obesity. Only 26.8% of students rated genetics or biological factors as very important. Lack of willpower was rated as less important than genetics or biological factors, but over 40% of students considered it to be at least a moderately important cause of obesity.

**Table 2:** Students’ beliefs about the causes of obesity (*n* = 71)

Causes of obesity		Percentage of respondents rating the importance of each to obesity				Mean	SD	Cronbach’s alpha
		1 Moderately Important	2 Somewhat important	3 Moderately important	4 Very important			
Within a person’s control								
A	Unhealthy diet (e.g., sweetened beverages, fast food, etc.)	0	1.4	36.6	62.0	3.61	.520	0.742
B	Physical inactivity	0	2.8	40.8	56.3	3.54	.556	
C	Overeating	0	4.2	43.7	52.1	3.48	.582	
D	Lack of willpower	11.4	44.3	34.3	10.0	2.43	.827	
Outside a person’s control								
E	Poor nutritional knowledge	1.4	5.6	40.8	52.1	3.44	.670	0.706
F	Lack of access to healthy foods	0	11.3	39.4	49.3	3.38	.684	
G	Psychological problems	0	9.9	43.7	46.5	3.37	.660	
H	Metabolic defect/ Endocrine disorder	0	15.5	42.3	42.3	3.27	.716	
I	Genetics or biological factors	1.4	19.7	52.1	26.8	3.04	.726	

Significant differences: I < A, B, C, E. D < A, B, C, E, F, G, H, I

Significant differences were determined by one-way ANOVA followed by a Tukey’s test to correct for multiple comparisons, *p* < 0.05

Table 3 lists the survey items evaluating explicit personal discomfort and bias towards people with obesity. Twenty five percent of students somewhat or strongly agreed with the statement, “I feel uncomfortable when examining an obese patient.” Seventeen percent indicated that they “somewhat agree” or “strongly agree,” that obese individuals don’t make good decisions, 8% agreed that obese workers cannot be as successful as other workers, and 11% agreed that obese individuals are lazier than non-obese people.

**Table 3:** Attitudes towards people with obesity (*n* = 71)

Survey Question	Percentage of respondents rating how much they agree or disagree with each statement				Mean	SD	Cronbach’s alpha
	1 Strongly disagree	2 Somewhat disagree	3 Somewhat agree	4 Strongly agree			
Personal discomfort							
I have negative reactions towards the appearance of obese patients.	36.6	45.1	16.9	1.4	1.83	.756	0.687
I feel uncomfortable when examining an obese patient.	38.0	35.2	23.9	2.8	1.92	.858	
Obesity Bias							
Obese individuals don’t make good decisions.	38.0	45.1	15.5	1.4	1.80	.749	0.767
Obese workers cannot be as successful as other workers.	59.2	32.4	5.6	2.8	1.52	.734	
Obese individuals are lazier than non-obese people.	53.5	35.2	9.9	1.4	1.59	.729	

Survey questions elicited student attitudes towards people with obesity on a 4-point Likert-type Scale (1, strongly disagree; 2, somewhat disagree; 3, somewhat agree; 4, strongly agree)

Survey item results were organized into five factor families with satisfactory internal consistency (Table 4) for further correlation analyses. A higher “external attribution score” indicates belief that external factors rather than internal factors are the most important contributors to obesity, and this score was significantly negatively correlated with obesity bias (Spearman’s correlation coefficient, − 0.447) (Table 4).

**Table 4:**
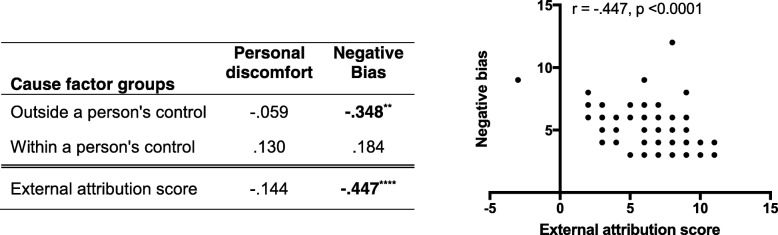
Correlation of beliefs about obesity causes with obesity bias (*n* = 71)

** *p* < 0.01 (2-tailed); **** *p* < 0.0001 (2-tailed)

Bivariate two-tailed Spearman’s correlations were calculated to determine associations between bias measures

**Table 5:** Correlation of OSCE performance with beliefs and attitudes (*n* = 71)

		Causes of obesity			Bias	
OSCE Assessment Domain		Outside a person’s control	Within a person’s control	External attribution score	Personal discomfort	Negative Bias
Information gathering		−.113	−.134	−.003	−.099	−.092
Relationship Building		.005	−.097	.079	−.038	.013
Educate		.116	−.151	**.266** ^*^	−.039	−.178
5As counseling strategy		.048	**−.235** ^*^	**.276** ^*^	−.191	−.169
Patient activation		−.090	−.177	.027	−.045	−.121
Overall Recommendation		−.041	**−.270** ^*^	.191	−.083	−.046
Total OSCE score		−.002	**−.257** ^*^	.231	−.144	−.129

Bivariate two-tailed Spearman’s correlations were calculated to determine associations between OSCE performance domains versus their beliefs and attitudes about people with obesity. *, *p* < 0.05 (2-tailed)

### Clinical skills in obesity OSCE case

Students were graded by standardized patients in the communication domains of information gathering, relationship building, utilizing the 5A’s counseling strategy, patient activation, and overall performance. Each domain consisted of specific skill parameters that were grouped together for analysis (Table 5). Believing obesity was within a person’s control was negatively correlated with students’ ability to utilize the 5A’s counseling strategy during the standardized patient encounter, and negatively correlated with student’s overall professionalism and recommendation. Though personal discomfort and negative bias trended toward negative correlations with students’ ability to counsel patients effectively, they did not reach statistical significance in our sample.

### Regression analysis

To look at the influence of students’ attitudes on obesity counseling while controlling for general communication skills, we generated a regression model with the 5A’s counseling strategy score as the dependent variable, with attitude factors (External Attribution score, personal discomfort, and negative bias) and the OSCE “information gathering” factor as independent variables. Our model suggests that attributing obesity to external causes contributes a modest amount to explaining the variation in ability of students to counsel patients effectively (standardized beta coefficient 0.23, *p* = 0.05; R2 (total variance explained) = 26.2%).

## Discussion

In this study, we found that third year medical students rate controllable factors such as unhealthy diet, physical inactivity, and overeating as more important contributors to obesity than genetics or biological factors. This is consistent with several previous studies that have surveyed health providers about their beliefs about obesity and shown that many do not rank heritability as an important cause of obesity [22, 30, 31, 40, 44–46]. In a survey of US primary care physicians, genetic factors ranked below physical inactivity, overeating, and high fat diet as important causes of obesity. More than 30% viewed patients with obesity as weak-willed, sloppy, or lazy, over 50% viewed them as awkward, unattractive, ugly, and noncompliant, and only 50% of physicians rated genetic factors as a very important cause of obesity [31]. More recently, a survey of US-based cardiologists, endocrinologists, and primary care providers showed that about half agreed that obesity is a due to a lack of self-control [47].

Previous studies have found that attributing obesity to external uncontrollable factors was negatively correlated with obesity bias in general populations in several countries [15], but this has not yet been shown in healthcare providers. We found that this relationship between belief about the causes of obesity and the extent of anti-fat stigma can be identified in medical students. Of particular note, we found that believing that obesity is caused by factors outside a person’s control was positively correlated with proficiency in obesity counseling skills. Despite the numerous variables that affect how well a medical student communicates with patients (i.e. personality factors and level of preparedness for the OSCE exam), we still found that attributing obesity to external causes clearly contributes to the ability of students to counsel patients effectively. To our knowledge, this is the first study that uses a standardized scoring method to determine whether there is a correlation between beliefs about obesity and the ability of medical providers to communicate with and counsel patients with overweight effectively.

Educating people about the uncontrollable causes of obesity can reduce both implicit and explicit anti-fat bias [48, 49]. We cannot delineate cause and effect in this correlative study, or whether there is a confounding factor linking the belief that obesity has extrinsic causes with decreased anti-fat prejudice. However, it is likely that understanding the genetic and biological pathophysiology of obesity can reduce bias and may, in turn, make physicians more sympathetic toward patients with obesity, reducing patient stigmatization. Patients with obesity often feel stigmatized and judged by physicians, making them less likely to seek healthcare when appropriate [21, 25], and this stigmatization is actually counterproductive to weight loss goals [27–29]. Although maintaining drastic weight loss is exceedingly difficult [50], losing even 3–5% of body weight improves many health indicators [51, 52]. Additionally, structured weight loss programs can help individuals maintain this type of weight loss [53–55]. Thus, even though our current medical or lifestyle-based interventions rarely are able to fully cure obesity, behavioral interventions, encouraged by skilled and empathic providers, are still worthwhile.

Limitations of our study included the relatively small sample size within a single medical school, which limits the generalizability of the results. We also acknowledge the likelihood of having primed students to be aware of social desirability and other biases during their OSCE and within the survey, particularly since students had just completed a nutrition and obesity curriculum unit. Students may not have fully disclosed their beliefs, even in an anonymous survey. If this had any effect, however, it would be to diminish the variance in survey responses and OSCE performances, making correlations less pronounced. Thus, our results are likely a more conservative estimate of the effects of obesity beliefs on counseling proficiency.

## Conclusion

Our study is a first step in evaluating the effect of beliefs about obesity on provider care. We demonstrated in the medical student population that placing more weight on uncontrollable causes of obesity is correlated with decreased anti-fat bias, and is positively correlated with obesity counseling skills. Our findings suggest that educating healthcare providers on the biological causes of obesity could help reduce bias and improve care for both weight-related and unrelated health problems. Research about the most effective methods for teaching the basis of obesity and reducing bias is sparse, however [48, 56, 57], and more studies are needed to identify best practices.

## Abbreviations

ICI: Interclerkship intensive
OSCE: Objective Structured Clinical Experience
SP: Standardized patient

## References

[1] Ogden CL, Carroll MD, Fryar CD, Flegal KM (2015). Prevalence of obesity among adults and youth: United States, 2011-2014. NCHS data brief.

[2] Taylor P, Funk C, Craighill P (2006). Americans see weight problems everywhere but in the mirror.

[3] Sorensen TI, Price RA, Stunkard AJ, Schulsinger F (1989). Genetics of obesity in adult adoptees and their biological siblings. BMJ (Clinical research ed).

[4] Maes HH, Neale MC, Eaves LJ (1997). Genetic and environmental factors in relative body weight and human adiposity. Behav Genet.

[5] Stunkard AJ, Sorensen TI, Hanis C, Teasdale TW, Chakraborty R, Schull WJ, Schulsinger F (1986). An adoption study of human obesity. N Engl J Med.

[6] Price RA, Cadoret RJ, Stunkard AJ, Troughton E (1987). Genetic contributions to human fatness: an adoption study. Am J Psychiatry.

[7] Stunkard AJ, Harris JR, Pedersen NL, McClearn GE (1990). The body-mass index of twins who have been reared apart. N Engl J Med.

[8] Stunkard AJ, Foch TT, Hrubec Z (1986). A twin study of human obesity. Jama.

[9] Allison DB, Kaprio J, Korkeila M, Koskenvuo M, Neale MC, Hayakawa K (1996). The heritability of body mass index among an international sample of monozygotic twins reared apart. Int J Obes Relat Metab Disord.

[10] Herrera BM, Lindgren CM (2010). The genetics of obesity. Curr Diab Rep.

[11] Bjorntorp P (1993). Visceral obesity: a “civilization syndrome”. Obes Res.

[12] Tamashiro KL (2011). Metabolic syndrome: links to social stress and socioeconomic status. Ann N Y Acad Sci.

[13] French SA, Story M, Jeffery RW (2001). Environmental influences on eating and physical activity. Annu Rev Public Health.

[14] Puhl RM, Heuer CA (2009). The stigma of obesity: a review and update. Obesity (Silver Spring, Md).

[15] Puhl RM, Latner JD, O'Brien K, Luedicke J, Danielsdottir S, Forhan M (2015). A multinational examination of weight bias: predictors of anti-fat attitudes across four countries. Int J Obes (Lond).

[16] Allison DB, Basile VC, Yuker HE (1991). The measurement of attitudes toward and beliefs about obese persons. Int J Eat Disord.

[17] Persky S, Eccleston CP (2011). Medical student bias and care recommendations for an obese versus non-obese virtual patient. Int J Obes (Lond).

[18] Pantenburg B, Sikorski C, Luppa M, Schomerus G, Konig HH, Werner P, Riedel-Heller SG (2012). Medical students’ attitudes towards overweight and obesity. PLoS One.

[19] Miller DP, Spangler JG, Vitolins MZ, Davis SW, Ip EH, Marion GS, Crandall SJ (2013). Are medical students aware of their anti-obesity bias?. Acad Med.

[20] Crandall CS, Moriarty D (1995). Physical illness stigma and social rejection. Br J Soc Psychol.

[21] Puhl RM, Heuer CA (2010). Obesity stigma: important considerations for public health. Am J Public Health.

[22] Hebl MR, Xu J (2001). Weighing the care: physicians’ reactions to the size of a patient. Int J Obes Relat Metab Disord.

[23] Adams CH, Smith NJ, Wilbur DC, Grady KE (1993). The relationship of obesity to the frequency of pelvic examinations: do physician and patient attitudes make a difference?. Women & health.

[24] Drury CA, Louis M (2002). Exploring the association between body weight, stigma of obesity, and health care avoidance. J Am Acad Nurse Pract.

[25] Amy NK, Aalborg A, Lyons P, Keranen L (2006). Barriers to routine gynecological cancer screening for White and African-American obese women. Int J Obes (Lond).

[26] Friedman KE, Ashmore JA, Applegate KL (2008). Recent experiences of weight-based stigmatization in a weight loss surgery population: psychological and behavioral correlates. Obesity (Silver Spring, Md).

[27] Hansson LM, Rasmussen F (2014). Association between perceived health care stigmatization and BMI change. Obes Facts.

[28] Sutin AR, Terracciano A (2013). Perceived weight discrimination and obesity. PLoS One.

[29] Jackson SE, Beeken RJ, Wardle J (2014). Perceived weight discrimination and changes in weight, waist circumference, and weight status. Obesity (Silver Spring, Md).

[30] Huizinga MM, Cooper LA, Bleich SN, Clark JM, Beach MC (2009). Physician respect for patients with obesity. J Gen Intern Med.

[31] Foster GD, Wadden TA, Makris AP, Davidson D, Sanderson RS, Allison DB, Kessler A (2003). Primary care physicians’ attitudes about obesity and its treatment. Obes Res.

[32] Sabin JA, Marini M, Nosek BA (2012). Implicit and explicit anti-fat bias among a large sample of medical doctors by BMI, race/ethnicity and gender. PLoS One.

[33] Warner CH, Warner CM, Morganstein J, Appenzeller GN, Rachal J, Grieger T (2008). Military family physician attitudes toward treating obesity. Mil Med.

[34] Puhl RM, Brownell KD (2006). Confronting and coping with weight stigma: an investigation of overweight and obese adults. Obesity (Silver Spring, Md).

[35] Anderson DA, Wadden TA (2004). Bariatric surgery patients’ views of their physicians’ weight-related attitudes and practices. Obes Res.

[36] Unrod M, Smith M, Spring B, DePue J, Redd W, Winkel G (2007). Randomized controlled trial of a computer-based, tailored intervention to increase smoking cessation counseling by primary care physicians. J Gen Intern Med.

[37] Serdula MK, Khan LK, Dietz WH (2003). Weight loss counseling revisited. Jama.

[38] Fitzpatrick SL, Wischenka D, Appelhans BM, Pbert L, Wang M, Wilson DK, Pagoto SL (2016). An Evidence-based Guide for Obesity Treatment in Primary Care. Am J Med.

[39] Kushner RF, Zeiss DM, Feinglass JM, Yelen M (2014). An obesity educational intervention for medical students addressing weight bias and communication skills using standardized patients. BMC Med Educ.

[40] Jay M, Kalet A, Ark T, McMacken M, Messito MJ, Richter R, Schlair S, Sherman S, Zabar S, Gillespie C (2009). Physicians’ attitudes about obesity and their associations with competency and specialty: a cross-sectional study. BMC Health Serv Res.

[41] Schlair S, Hanley K, Gillespie C, Disney L, Kalet A, Darby PC, Frank E, Spencer E, Harris J, Jay M (2012). How medical students’ behaviors and attitudes affect the impact of a brief curriculum on nutrition counseling. J Nutr Educ Behav.

[42] Jelalian E, Boergers J, Alday CS, Frank R (2003). Survey of physician attitudes and practices related to pediatric obesity. Clin Pediatr.

[43] Block JP, DeSalvo KB, Fisher WP (2003). Are physicians equipped to address the obesity epidemic? Knowledge and attitudes of internal medicine residents. Prev Med.

[44] Teachman BA, Brownell KD (2001). Implicit anti-fat bias among health professionals: is anyone immune?. Int J Obes Relat Metab Disord.

[45] Schwartz MB, Chambliss HO, Brownell KD, Blair SN, Billington C (2003). Weight bias among health professionals specializing in obesity. Obes Res.

[46] Ferrante JM, Piasecki AK, Ohman-Strickland PA, Crabtree BF (2009). Family physicians’ practices and attitudes regarding care of extremely obese patients. Obesity (Silver Spring, Md).

[47] Glauser TA, Roepke N, Stevenin B, Dubois AM, Ahn SM (2015). Physician knowledge about and perceptions of obesity management. Obes Res Clin Pract.

[48] O'Brien KS, Puhl RM, Latner JD, Mir AS, Hunter JA (2010). Reducing anti-fat prejudice in preservice health students: a randomized trial. Obesity (Silver Spring, Md).

[49] Puhl RM, Schwartz MB, Brownell KD (2005). Impact of perceived consensus on stereotypes about obese people: a new approach for reducing bias. Health Psychol.

[50] Fothergill E, Guo J, Howard L, Kerns JC, Knuth ND, Brychta R, Chen KY, Skarulis MC, Walter M, Walter PJ (2016). Persistent metabolic adaptation 6 years after “the biggest loser” competition. Obesity.

[51] Jensen MD, Ryan DH, Apovian CM, Ard JD, Comuzzie AG, Donato KA, Hu FB, Hubbard VS, Jakicic JM, Kushner RF (2014). 2013 AHA/ACC/TOS guideline for the management of overweight and obesity in adults: a report of the American College of Cardiology/American Heart Association task force on practice guidelines and the Obesity Society. J Am Coll Cardiol.

[52] Magkos F, Fraterrigo G, Yoshino J, Luecking C, Kirbach K, Kelly SC, de Las FL, He S, Okunade AL, Patterson BW (2016). Effects of moderate and subsequent progressive weight loss on metabolic function and adipose tissue biology in humans with obesity. Cell Metab.

[53] Al-Khudairy L, Loveman E, Colquitt JL, Mead E, Johnson RE, Fraser H, Olajide J, Murphy M, Velho RM, O'Malley C (2017). Diet, physical activity and behavioural interventions for the treatment of overweight or obese adolescents aged 12 to 17 years. Cochrane Database Syst Rev.

[54] Samdal GB, Eide GE, Barth T, Williams G, Meland E (2017). Effective behaviour change techniques for physical activity and healthy eating in overweight and obese adults; systematic review and meta-regression analyses. Int J Behav Nutr Phys Act.

[55] Anderson JW, Konz EC, Frederich RC, Wood CL (2001). Long-term weight-loss maintenance: a meta-analysis of US studies. Am J Clin Nutr.

[56] Vitolins MZ, Crandall S, Miller D, Ip E, Marion G, Spangler JG (2012). Obesity educational interventions in U.S. medical schools: a systematic review and identified gaps. Teach Learn Med.

[57] Alberga AS, Pickering BJ, Alix Hayden K, Ball GD, Edwards A, Jelinski S, Nutter S, Oddie S, Sharma AM, Russell-Mayhew S (2016). Weight bias reduction in health professionals: a systematic review. Clin Obes.

